# An International Reference Consensus Genetic Map with 897 Marker Loci Based on 11 Mapping Populations for Tetraploid Groundnut (*Arachis hypogaea* L.)

**DOI:** 10.1371/journal.pone.0041213

**Published:** 2012-07-18

**Authors:** Bhimana Gautami, Daniel Foncéka, Manish K. Pandey, Márcio C. Moretzsohn, Venkataswamy Sujay, Hongde Qin, Yanbin Hong, Issa Faye, Xiaoping Chen, Amindala BhanuPrakash, Trushar M. Shah, Makanahally V. C. Gowda, Shyam N. Nigam, Xuanqiang Liang, Dave A. Hoisington, Baozhu Guo, David J. Bertioli, Jean-Francois Rami, Rajeev K. Varshney

**Affiliations:** 1 Center of Excellence in Genomics, International Crops Research Institute for the Semi-Arid Tropics (ICRISAT), Patancheru, India; 2 UMR Développement et Amélioration des plantes, Centre de coopération Internationale en Recherche Agronomique pour le Développement (CIRAD), Montpellier, France; 3 Department of Plant Pathology, University of Georgia (UGA), Tifton, Georgia, United States of America; 4 Crop Protection and Management Research Unit, USDA-Agricultural Research Service, Tifton, Georgia, United States of America; 5 Plant Genetics Lab, EMBRAPA Genetic Resources and Biotechnology, Brasilia, Brazil; 6 Department of Genetics and Plant Breeding, University of Agricultural Sciences (UAS-D), Dharwad, India; 7 Cash Crop Research Institute, Hubei Academy of Agricultural Sciences (HAAS), Wuhan, Hubei, China; 8 Crops Research Institute, Guangdong Academy of Agricultural Sciences (GAAS), Guangzhou, Guangdong, China; 9 Centre National de Recherche Agronomique (CNRA), Institut Sénégalais de Recherches Agricoles (ISRA), Bambey, Sénégal; 10 Genetics Department, University of Brasilia, Brasilia, Brazil; 11 Theme- Comparative and Applied Genomics, CGIAR Generation Challenge Programme (GCP), CIMMYT, Mexico, Mexico; University of New England, Australia

## Abstract

Only a few genetic maps based on recombinant inbred line (RIL) and backcross (BC) populations have been developed for tetraploid groundnut. The marker density, however, is not very satisfactory especially in the context of large genome size (2800 Mb/1C) and 20 linkage groups (LGs). Therefore, using marker segregation data for 10 RILs and one BC population from the international groundnut community, with the help of common markers across different populations, a reference consensus genetic map has been developed. This map is comprised of 897 marker loci including 895 simple sequence repeat (SSR) and 2 cleaved amplified polymorphic sequence (CAPS) loci distributed on 20 LGs (a01–a10 and b01–b10) spanning a map distance of 3, 863.6 cM with an average map density of 4.4 cM. The highest numbers of markers (70) were integrated on a01 and the least number of markers (21) on b09. The marker density, however, was lowest (6.4 cM) on a08 and highest (2.5 cM) on a01. The reference consensus map has been divided into 20 cM long 203 BINs. These BINs carry 1 (a10_02, a10_08 and a10_09) to 20 (a10_04) loci with an average of 4 marker loci per BIN. Although the polymorphism information content (PIC) value was available for 526 markers in 190 BINs, 36 and 111 BINs have at least one marker with >0.70 and >0.50 PIC values, respectively. This information will be useful for selecting highly informative and uniformly distributed markers for developing new genetic maps, background selection and diversity analysis. Most importantly, this reference consensus map will serve as a reliable reference for aligning new genetic and physical maps, performing QTL analysis in a multi-populations design, evaluating the genetic background effect on QTL expression, and serving other genetic and molecular breeding activities in groundnut.

## Introduction

Dense genetic linkage maps are cornerstones for wide spectrum of genetics and breeding applications such as linkage mapping or association analysis based trait mapping, marker-assisted breeding, map-based cloning and physical map alignment. In general, it is possible to map only limited number of molecular markers in a given mapping population due to polymorphism constraints. As a result, several mapping populations are used for developing different genetic maps so that maximum number of marker loci available are mapped in the given crop species. Subsequently, with an objective to increase the number of mapped marker loci, genetic maps developed for different mapping populations are used for developing a consensus map. As compared to individual genetic maps, consensus maps offer several advantages such as: (i) mapping of a large number of marker loci onto a single map, (ii) determining relative position of common markers across the mapping populations, (iii) determining stability of marker locus position across the genome, (iv) provides evidence for chromosomal rearrangements [1], [2], gene duplication [2], [3] and assists in the assignment of linkage groups to chromosome [1], (v) provides the basic information for comparative genomic studies among related species and subspecies [2]–[4] and (vi) provides genetic information for greater genomic coverage [5]. Because of above mentioned features, consensus genetic maps have been developed in many crop species like maize [6], [7], wheat [8] barley, [9], [10], soybean [11], [12] and pigeonpea [13].

Groundnut or peanut (*Arachis hypogaea* L.), an economically important oil seed crop, is cultivated mostly in semi-arid regions of the world. It is an allotetraploid (2*n* = 4*x* = 40) with a large genome size 2800 Mb/1C. Based on the origin complexity such as polyploidy nature, narrow genetic base with very low DNA polymorphism in cultivated tetraploid groundnuts, initially genetic maps were developed for AA- genome [14]–[16] and BB- genome [17], [18]. Only recently a few mapping populations have been used for developing the genetic maps for cultivated groundnut species [19]–[22] or based on cross of cultivated and synthetic tetraploid groundnut species [23]. In some cases, consensus genetic maps based on two or three mapping populations have also been developed [24]–[27]. The most dense consensus genetic map developed so far is based on two mapping populations and is comprised of 324 SSR loci [27]. However because of availability of >4000 SSR markers in *Arachis* species [28], international *Arachis* community has been striving towards developing a consensus genetic map compiling a maximum number of genetic markers especially when efforts have been initiated to sequence the genome of *Arachis* species (http://www.peanutbioscience.com/peanutgenomeproject.html).

Keeping in view of above, this article reports assembling of SSR marker genotyping data for 11 mapping populations including 10 recombinant inbred lines (RILs) and one backcross (BC) population. These genotyping data have been used to develop a consensus genetic map with 895 SSR marker loci and 2 CAPS loci. For enhancing the utility of the consensus genetic map, the map has been divided into 20 cM long 203 BINs and the polymorphism information content (PIC) values for the markers, wherever possible, present in these BINs have also been presented.

## Results

### High-quality Marker Segregation Data

Marker segregation data were assembled for a total of 1961 markers ranging from 64 markers (RIL-8) to 339 markers (BC-1) per population (Table 1). A chi-square test was performed on marker genotyping data for individual mapping population to test the null hypothesis of segregation ratios of 1∶1 at the threshold of *p* = 0.05. A variable percentage of distorted markers ranging from 3.45% (RIL-8) to 52.34% (RIL-2) were observed for individual mapping populations.

**Table 1:** Source of marker data used for constructing the reference consensus genetic map.

Mapping populations	Population type	Populationsize	Genotyping dataassembled (no. of loci)	Trait segregation
*International Crops Research Institute for the Semi-Arid Tropics (ICRISAT), Patancheru, India*				
TAG 24 × ICGV 86031 (RIL-1)	RIL (F_8_)	318	211	Drought tolerance related traits
ICGS 76 × CSMG 84–1 (RIL-2)	RIL (F_9_)	176	128	Drought tolerance related traits
ICGS 44 × ICGS 76 (RIL-3)	RIL (F_8_)	188	87	Drought tolerance related traits
*University of Agricultural Sciences-Dharwad (UAS-D), Dharwad, India*				
TAG 24 × GPBD4 (RIL-4)	RIL (F_7_)	266	209	Late leaf spot and rust resistance
TG 26 × GPBD 4 (RIL-5)	RIL (F_7_)	146	209	Late leaf spot and rust resistance
*Guangdong Academy of Agricultural Sciences (GAAS), China*				
Yueyou 13 × Zhen Zhuhei (RIL-6)	RIL (F_4∶6_)	142	146	Protein content
Yueyou 13 × FU 95–5 (RIL-7)	RIL (F_4∶6_)	84	124	Oil content
Yueyou 13 × J 11 (RIL-8)	RIL (F_4∶6_)	136	64	Resistance to *Aspergillus flavus* and aflatoxincontamination
*University of Georgia (UGA), Tifton, USA*				
Tifrunner × GT-C20 (RIL-9)	RIL (F_2∶6_)	248	261	Tomato spotted wilt virus (TSWV) resistanceand several agronomic traits
SunOleic 97R × NC94022 (RIL-10)	RIL (F_2∶6_)	352	197	Tomato spotted wilt virus (TSWV) resistanceand several agronomic traits
*Centre de coopération Internationale en Recherche Agronomique pour le Développement (CIRAD), Montpellier, France*				
Fleur11 × AiAd (synthetic amphidiploid) (BC-1)	BC_1_F_1_	88	339	Several agronomic traits

### Individual or Component Genetic Maps

The genotyping data obtained on 11 mapping populations (1961 markers) were used for constructing the component genetic maps for the respective mapping population using MAPMAKER/EXP V 3.0 [29]. All developed component genetic maps can be visualized in CMap database at http://cmap.icrisat.ac.in/cmap/sm/gn/gautami/. The numbers of marker loci ranged from 46 (RIL-8) to 332 (BC-1) per component genetic maps for different mapping populations. Genetic map distance covered from 357.4 cM (RIL-8) to 2208.2 cM (RIL-2) with a range of map density from 2.5 cM (BC-1) to 18.6 cM (RIL-2) (Table 2). As several markers integrated into component maps have segregation distortion, the linkage group (LG)-wise segregation pattern of markers in each mapping population has been shown in Figure S1.

**Table 2:** Features of the component and reference consensus genetic maps.

Maps	Linkage groups	Mappedloci	Map length(cM)	Map density(cM)	Inter-locus gap distance (cM)	References
RIL-1	22	191	1785.4	9.4	9.4	Varshney et al. 2009b, Ravi et al. 2011
RIL-2	20	119	2208.2	18.6	18.7	Gautami et al. 2012
RIL-3	15	82	831.4	10.1	10.3	Gautami et al. 2012
RIL-4	20	188	1922.4	10.2	10.3	Khedikar et al. 2010, Sujay et al. 2012
RIL-5	21	181	1963.0	10.8	10.9	Sarvamangala et al. 2011, Sujay et al. 2012
RIL-6	19	133	793.1	6.0	6.1	Hong et al. 2010
RIL-7	21	109	503.1	4.6	4.7	Hong et al. 2010
RIL-8	13	46	357.4	7.7	7.9	Hong et al. 2010
RIL-9	26	233	1304.9	5.6	5.6	Qin et al. 2012
RIL-10	22	193	917.5	5.3	5.4	Qin et al. 2012
BC-1	21	332	847.4	2.5	2.6	Foncéka et al. 2009
Reference consensusgenetic map	20	897	3863.6	4.4	4.5	–

### Reference Consensus Genetic Map

A reference consensus genetic map was constructed by integrating all 11 component genetic maps using common markers across different genetic maps using MergeMap program. While integrating component genetic maps, some discrepancies were observed in the names of markers for which genotyping data were available on more than one mapping population. However, to facilitate integration, uniformity in marker nomenclature was maintained for all the markers. For example, ‘pPGPseq xx’ and pPGSseqxx’ were changed to ‘seqxx’, and ‘XIPxx’ was changed to ‘IPAHMxx’ to maintain uniformity in names of marker loci.

Based on the common markers and the comparison between component genetic maps, most of the linkage groups were consistent among the individual maps with few exceptions which can be visually assessed from http://cmap.icrisat.ac.in/cmap/sm/gn/gautami/(also see Table S1). A total of 542 markers were unique markers i.e. mapped only in one mapping population, while the remaining 355 markers were common, i.e. they were mapped in at least two mapping populations (187 markers were common between two maps, 72 between three maps, for four maps 57 are common, 20 markers are common between 5 maps, between 6 maps 13 markers are common, 3 markers are common between 7 maps, 2 markers between 8 maps and 1 marker is common between 9 maps) and served as anchor points for the map integration (Table 3). The grouping of different LGs from component genetic maps to develop the consensus map were given in Table S2. Therefore, in the consensus genetic map, a total of 39.58% (355) markers are anchor markers present on all 20 LGs. The remaining 60.42% (542) markers are the markers which are unique to the individual mapping populations.

**Table 3:** Summary of number of loci common between genetic maps for different mapping populations.

S.No	Mapping population	No. of mapped loci	No. of mapped loci used in consensus map	Number of markers in common with *n* other mapping populations								
				*n* = 0	*n* = 1	*n* = 2	*n* = 3	*n* = 4	*n* = 5	*n* = 6	*n* = 7	*n* = 8
1	RIL-1	191	178	55	36	35	27	11	8	3	2	1
2	RIL-2	119	81	39	12	7	11	5	4	2	1	0
3	RIL-3	82	72	18	14	16	8	9	4	1	1	1
4	RIL-4	188	176	19	67	28	32	16	9	2	2	1
5	RIL-5	181	168	17	72	23	31	12	8	3	1	1
6	RIL-6	133	114	27	28	18	17	10	9	3	1	1
7	RIL-7	109	96	12	30	14	20	7	9	2	1	1
8	RIL-8	46	36	10	4	7	6	4	3	0	1	1
9	RIL-9	233	194	85	43	19	23	12	7	2	2	1
10	RIL-10	193	145	51	40	19	18	7	7	1	2	0
11	BC-1	332	324	209	28	30	35	7	10	2	2	1
	Total			542	187	72	57	20	13	3	2	1

Multiple segregating fragments (loci) identified with one SSR primer pair were designated with one lower case letter “a”, “b” or “c” as suffix with the name of marker. For example two loci mapped on LG_AhVII and LG_AhXVII by using the same marker (RIL-1), were designated as IPAHM108a and IPAHM108b loci (Table S1).

**Figure 1:**
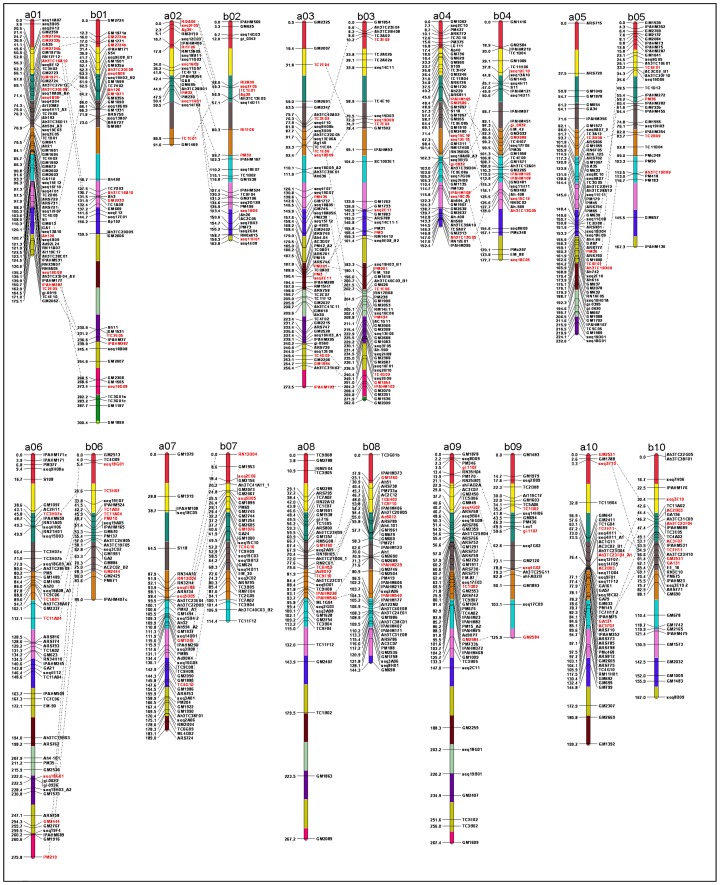
A microsatellite consensus genetic map comprising 897 marker loci based on 11 mapping populations. Markers are shown on *right* side of the LG while map distances are shown on the *left* side. Each LG has been divided into 203 BINs of 20 cM each. The homoeologous loci between the corresponding LGs in the reference consensus map are indicated in red colour.

Seventy homeologous loci were identified on “a” and “b” linkage groups (Figure 1), which facilitate the detection of ten homeologous pair named from a01 to a10 and b01 to b10 based on the same loci detected on the framework map (BC-1 in the present study) developed by Foncéka et al. [23]. Out of these 70 homeologous loci, 11 loci were located between the group a01–b01 and a03–b03, followed by eight loci between a02–b02 and a04–b04 and four loci between a09–b09. Except for the groups between a01–b01, a03–b03 and a04–b04 markers order and inter-loci map distance were well conserved between homeologous groups (Figure 1).

In some cases, the same marker mapped single locus on different linkage groups in different mapping populations, these loci were not considered as the same loci and were included as unique loci (with the same name) in the reference consensus genetic map. Twenty nine (26.13%) primer pairs detected duplicated non-homeologous loci between linkage groups (e.g., seq12F07 detected two loci, one on a01 and one on a10; IPAHM524 detected two loci, one on b02 and one on b06 and IPAHM171 detected three loci on a06, b01 and b08) (Figure 1, Table S1).

Although it was planned to include only SSR marker loci in the reference consensus genetic map, two CAPS (cleaved amplified polymorphic sequence) markers i.e., ahFAD2A and ahFAD2B, due to their association with high oleic acid to linoleic acid ratios (high O/L) [30], very important trait in groundnut, were also integrated in the reference consensus genetic map.

In summary, the reference consensus map is comprised of 895 SSR and 2 CAPS loci distributed over 20 LGs. Nomenclature of LGs in the reference consensus map was given in the same way as in the framework map (BC-1 in the present study) developed by Foncéka et al [23]. The map density in the reference consensus map ranged from 2.5 cM (a01) to 6.4 cM (a08) with an average of 4.3 cM per marker. The inter-locus gap distance ranged from 1.5 cM (a01) to 5.4 cM (a08), with a mean value of 4.5 cM per marker (Table 4). Among the 20 LGs, a01 possess maximum marker loci with 70 loci followed by a03, a05 and b03 with 65, 61 and 60 loci respectively, while a02 and b09 have only 23 and 21 loci, respectively (Figure 1, Table 4). The low number of SSR loci mapped on a02 and b10 may be related to the lack of polymorphism on these two LGs. For example, the consensus LG a02 is built with seven LGs of the different component genetic maps, among which four LGs have only two mapped loci. For these small LGs additional genetic markers are needed for increasing the map density.

In the consensus map, some gaps are observed on the distal ends of the a02, b02, a03, a05, b05, a08, a09, b09 and a10. Of the 897 mapped loci, 32.33% (290 loci) of the marker intervals were smaller than 1 cM while 41.14% (369 loci) marker intervals were between 1–5 cM, 15.94% (143 loci) between 5–10 cM, 7.36% (66 loci) between 10–20 cM, and 3.23% (29 loci) marker intervals were greater than 20 cM.

**Table 4:** Features of the reference consensus genetic map.

LGs	No. of mapped loci	Map distance (cM)	Map density (cM)	Inter-locus gap distance (cM)
a01	70	175.1	2.5	2.5
b01	51	300.4	6.0	6.0
a02	23	91.6	4.0	4.2
b02	30	162.8	5.4	5.6
a03	65	272.5	4.1	4.3
b03	60	282.0	4.7	4.8
a04	56	152.4	2.7	2.8
b04	42	177.7	4.2	4.3
a05	61	232.6	3.8	4.0
b05	33	167.3	5.1	5.2
a06	57	275.8	4.8	5.0
b06	24	99.0	4.1	4.3
a07	43	189.0	4.4	5.0
b07	34	114.4	3.4	3.5
a08	42	267.2	6.4	6.5
b08	47	144.3	3.1	3.1
a09	56	267.4	4.8	5.0
b09	21	125.9	6.0	6.3
a10	47	199.2	4.2	4.3
b10	35	167.0	4.8	5.0
Total	897	3863.6	–	–
Mean	45	193.2	4.4	4.5

### Added Value Features of the Reference Consensus Genetic Map

As SSR markers are the marker of choice in breeding applications, an attempt was made to understand the distribution of different SSR motifs as well as the polymorphism information content (PIC) values for these markers.

Out of 895 SSR loci integrated into the reference consensus map, information on repeat motifs was available for 788 SSR loci. Of the 788 SSRs, 612 SSR loci represent simple repeat motifs and 176 SSR loci contain compound repeat motifs. Among simple repeat motifs contained SSR loci, 375 SSR loci (47.58%) are comprised of di- (NN) repeats followed by 226 (28.70%) tri-nucleotides (NNN) repeats. The longer repeat classes, i.e. tetra- (NNNN, 8 loci) and hexa-nucleotide (NNNNNN, 3 loci) represented 1.39% of the SSR loci (Table S3). In the case of the compound repeats containing SSR loci, 93 loci were comprised of NN repeats and the remaining 83 loci comprised with mixed repeats.

Of the 897 mapped marker loci, the information on PIC values was available for 526 SSR marker loci from the studies in which the corresponding SSR loci were mapped (Table S3). Based on genotypes surveyed in those earlier studies, 144 marker loci have >0.50 PIC value. Majority of the loci (181) have 0.31–0.40 PIC value (Figure S2). Average PIC value of individual LGs varied from 0.55 (a02) to 0.81 (a01).

For making the consensus map more informative, an attempt has been made to divide the genetic map in 20 cM long BINs. As a result, the reference groundnut genetic map has a total of 203 BINs ranging from 5 (a02 and b06) to 16 (b01) with an average of 4 per linkage group. These BINs carry 1 (a10_02, a10_08 and a10_09) to 20 (a10_04) with an average of 4.41 marker per BIN. In terms of selecting highly informative SSR markers based on available PIC values, 36 BINs have at least one marker that has >0.70 PIC value and 111 BINs carry at least one marker with >0.50 PIC value. 166 BINs have the marker loci with <0.50 PIC value and 23 BINs do not have the information available on PIC values. 13 BINs do not have any marker.

Finally, for deciphering the relationships between LGs of the different component maps, we have identified a total of 58 genome specific SSR markers. These markers will be of great interest for subgenome assignment of SSR loci in cultivated x cultivated mapping studies. These markers could also be used in diversity analysis as they give access to the diversity at the diploid genome level allowing differentiating the structural heterozygosity linked to polyploidy from true heterozygosity.

### Relationships of the Reference Genetic Map and Component Maps

**Figure 2:**
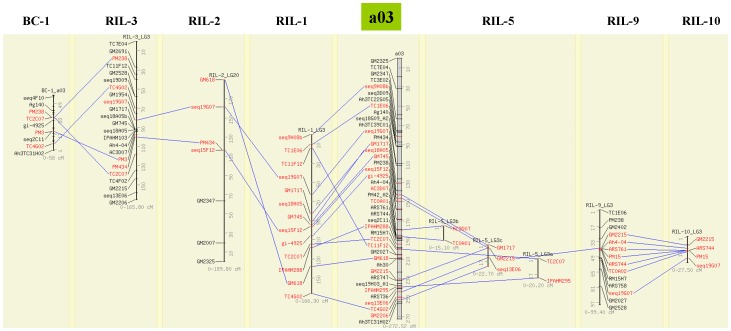
A marker based correspondence for a03 among reference consensus and individual genetic maps. Only common markers i.e. landmarks are included to visually asses the co-linearity of marker orders and marker positions. LGs are aligned together using comparative mapping program CMap version 1.01. Figure can also be accessed from http://cmap.icrisat.ac.in/cmap/sm/gn/gautami/.

As the reference map was developed based on the common marker loci mapped in the different component genetic maps using the same nomenclature of LGs, there was a good congruence except a few exceptions between marker orders and positions among component maps and the reference consensus map (http://cmap.icrisat.ac.in/cmap/sm/gn/gautami/and also see in Table S1). Comparison of a03 and b08 for all the component genetic maps and the reference consensus map, for example, has been shown in Figure 2.

### Comparison with Diploid Genetic Maps

**Figure 3:**
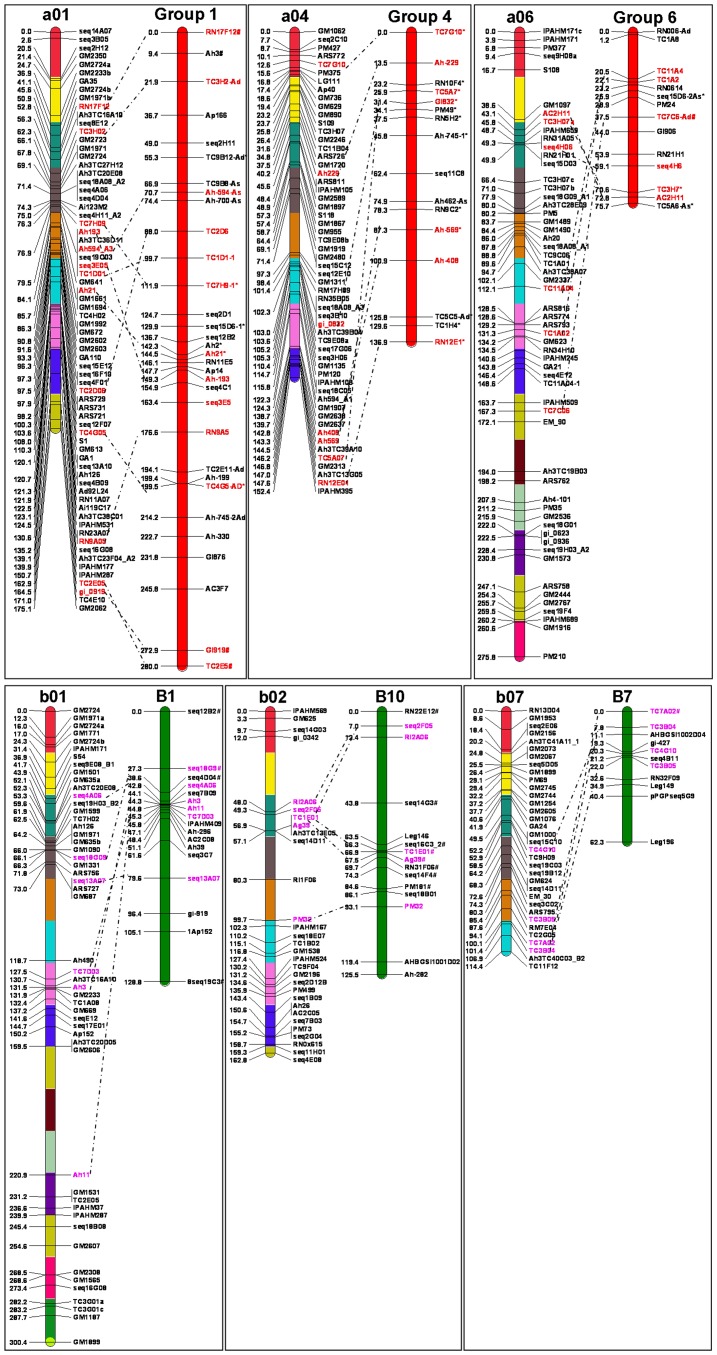
Comparison between the LGs of the reference consensus map and the diploid AA and BB maps. The LGs of the reference consensus map are represented as a01 to a10 and b01 to b10. The LGs of AA map are named as Group 1 to Group 11 and for BB map as B1 to B10 respectively (published by Moretzsohn et al 2005, 2009). The AA map was represented by a red bar and the BB map with green colour. The common markers between corresponding LGs in the reference consensus map and AA map are indicated in red colour and pink colour with BB map.

The results of the reference consensus genetic map were compared with the diploid AA and BB maps published earlier [15], [18]. The LGs of the reference consensus map in this study are named according to the LGs named in Foncéka et al. [23] (a01 to a10 and b01 to b10). In these maps, LGs of AA and BB genome map were named as Group 1 to Group 11 and B1 to B10 respectively. The synteny study between the reference consensus map and AA map assessed 68 common SSR marker loci and between BB map assessed 43 common SSR marker loci (Table S4). However, for all the ten LGs of the present constructed reference consensus genetic maps, overall good collinerity was observed for the corresponding LGs of the two diploid maps, with a few exceptions in some marker positions. The salient features of the comparison of the reference consensus genetic map with AA and BB maps for six LGs each are shown in the Figure 3. The number of common SSR marker loci per homologous LGs varied between 2 and 10 with AA map and with BB map between 1 and 9.

## Discussion

Significant progress has been made during last decade in high throughput genotyping and various linkage mapping tools to place a large number of marker loci on genetic maps in several crop species [8], [13], [31]–[33]. In the case of tetraploid groundnut, genetic mapping efforts have been initiated only recently and few genetic maps with 46–332 marker loci have been developed [34]. To enhance the marker density, a few consensus maps have also been developed using 2–3 mapping populations and the mapped marker loci on these maps has not gone beyond 324 loci. The major objective of this study, therefore, was the development of a dense consensus genetic map that can be used as a reference map by the international groundnut community.

Dense genetic maps can be developed mainly by using two approaches: (a) map maximum number of marker loci using highly diverse population, (b) merge available genetic maps using common markers mapped across the populations. While the first approach is quite challenging and laborious but precise, the second approach was used in the present study. In this context, segregation data for a total of 1961 marker loci generated for 11 (10 RIL and 1 BC) populations were assembled from different organizations. As a first step, component genetic maps were developed for all 11 populations. While comparing the component genetics maps developed in this study with the ones published by the source laboratory, all mapped marker loci could not be integrated into component genetic maps in this study. One of the main reasons for this may be use of a stringent and common approach to develop all the component genetic maps.

Building a consensus map is not possible without common or bridge loci present on each LG [9]. A bridge marker was considered as such when it had an identical name and should have a similar position in different mapping populations that are underpinned. Markers with the same name that mapped to different positions in different populations were not considered to be common or bridge markers. However a minimum of three common markers per linkage group should be considered while, in the present study, at least one common marker per LG is also taken into consideration in some LGs because of availability of lower number of markers in some LGs.

During the process of construction of consensus map, the major emphasis was given towards obtaining a general order and distance because as a known fact, groundnut is polyploid with a large genome size (2800 Mb/C), and has a narrow genetic base with very low DNA polymorphism. Slight discrepancies in marker orders as well as positions observed in some LGs (http://cmap.icrisat.ac.in/cmap/sm/gn/gautami/and Table S1) among different component genetic maps may be due to (i) different mapping population sizes used (ii) different type of mapping populations used and (iii) genotyping errors [35] or sometimes these small differences might be due to mapping- imprecision rather than real rearrangements.

Developed consensus map integrates a total of 897 marker loci including 895 SSR and 2 CAPS loci with an average map density of 4.3 cM. This map is the most dense and community map and, therefore, is proposed as a reference consensus map. Despite of dense placing of markers on various LGs, some gaps were observed on the distal ends of some LGs e.g. a02, b02, a03, a05, b05, a08, a09, b09 and a10. These regions may be high recombination prone regions and some of them were also observed in other mapping studies also [19], [21], [24]–[27]. Another reason for these gaps may be due to under-representation or deficiency of marker loci from these genomic regions in the dataset used for developing the reference consensus map [9], [13], [19], [21].

In present mapping protocol, the homologous LGs taking into consideration of homeologous relationship were used to generate consensus map LGs one at a time using MergeMap to establish marker orders (see materials and methods). Therefore, the marker orders in the consensus map are consistent throughout most of the linkage groups with few exceptions where the marker orders are in opposite orientation. Moreover, maximum markers were mapped onto the consensus map in their original orders similar to the individual maps, but small number of markers were joined with order changes, which could be caused by computational variation resulting from (i) recombination heterogeneity between different populations, (ii) weak linkages existing in the various LGs of maps, (iii) missing or poor quality data, (iv) different mapping programmes being used for constructing the individuals and the consensus maps and, (v) different thresholds statistics being applied for creating the consensus map and the original maps [36].

While utmost precautions were taken in preparing this consensus map, there could be some disagreement in order of closely linked markers between the individual maps within some LGs intervals. Such a disagreement may be due to the quality as well as the quantity and distribution along the LGs of the bridge (common) markers used for preparing the consensus map, or to mapping populations, algorithm and stringency criteria of computer programme [9], [24], [36]. For example, the mapping populations from which the consensus map has been prepared have different numbers and different types of progeny lines. In smaller populations, the chance that informative recombinant progeny lines are present in the population to accurately position markers is lower than in larger populations [9], [36]. Further, even for a given mapping population, different markers were mapped using different subsets of progeny lines in different laboratories. Therefore, the users of the consensus SSR map must consider that the marker order is conditioned by several factors like the progeny lines used and the position of cross over along chromosome within the progeny lines. The precise fine markers order may slightly differ in other population and users may need to verify the order of closely linked markers in their mapping and breeding populations.

This reference consensus map integrated almost all types of SSR motifs, however di- and tri-nucleotide microsatellites at 47.58% and 28.70%, respectively, are present in higher proportions than the compound (22.33%) and other types of SSRs (1.39%). The underlying reason may be that the majority of SSR loci integrated in the consensus map were derived from the genomic DNA libraries that had been enriched for dinucleotide and trinucleotide SSR probes [28], [34]. Therefore, the availability of different types of SSR loci in a given region will facilitate selection of the SSR repeat motifs of choice in a particular region of interest. Availability of the primer sequences for a total of 885 SSR loci, approximately 90% of all loci integrated in the consensus map, at one place should accelerate the use of SSR markers in groundnut breeding activities. Similarly, the genotyping data has been made available for all the mapped SSR loci in the present study and this will allow the community to extend the dataset with their own data set in future.

Another feature of the developed reference consensus map is the defining of the groundnut genetic map in 203 BINs. Furthermore marker loci present in these BINs are associated with the PIC values. One marker from each of such BIN with higher PIC value has also been identified. Using this criteria, 36 BINs have been identified that have at least one marker with >0.70 PIC value and 111 BINs carry at least one marker >0.50 PIC value. This information will be very useful to select the genome-wide markers that will have higher probability of showing polymorphism in the parental genotypes of the mapping populations or germplasm collections to be analyzed. It is also important to mention that primer sequence information also has been provided here for 885 markers.

## Materials and Methods

### Assembling Marker Segregation Data

SSR marker segregation data available on ten recombinant inbred lines (RILs) and one backcross (BC) mapping populations were assembled from collaborators as mentioned in Table 1. The populations, for which marker segregation data were assembled, for the convenience of referring in this article, have been referred as RIL-1 to RIL-10 and BC-1.

Three mapping populations (RIL-1, RIL-2, and RIL-3), developed at ICRISAT, segregate for drought tolerance related traits [25], two mapping populations (RIL-4 and RIL-5), developed at UAS-Dharwad, segregate for foliar disease resistance [26] and two populations (RIL-9 and RIL-10), developed at UGA and HAAS, segregate for tomato spotted wilt virus (TSWV). In the case of RIL-6, RIL-7 and RIL-8, developed at GAAS, Yueyou 13 (Y13), a Spanish type with high yield was the common female parent. While the RIL-6 segregates for oil content, the RIL-7 segregates for protein content and the RIL-8 segregates for resistance to Aspergillus flavus and aflatoxin contamination [24]. The remaining BC-1 population (BC1F1) was developed using a wild tetraploid AABB amphidiploid (*A. ipaënsis* KG30076 × *A. duranensis* V14167), called AiAd [37] and a cultivated tetraploid AABB variety (Fleur 11). This population segregates for several agro-morphological and drought related traits [38].

In brief, the segregation data for 211 marker loci on RIL-1 [19], [21], 128 and 87 markers loci on RIL-2 and RIL-3, respectively [25] and 209 marker loci each on RIL-4 and RIL-5 populations [20], [22], [26] were obtained. RIL-6, RIL-7 and RIL-8 contributed marker data for 146, 124 and 64 marker loci respectively [24]. Segregation data were obtained for 261 and 193 marker loci on RIL-9 and RIL-10, respectively [27]. The lone BC-1 population contributed segregation data of maximum number (339) of marker loci [23]. Genotyping data as mentioned above have been provided in Table S5.

### Construction of Component Genetic Maps

Segregation data for 1961 markers obtained on all the 11 mapping populations were subjected to chi-square (χ2) test to examine distortion from the expected 1∶1 segregation using “Locus genotype frequency” function of JoinMap V 3.0 [39]. Individual or component genetic maps were constructed using MAPMAKER/EXP [29] and Kosambi mapping function [40] to assemble linkage groups by maximum-likelihood for respective mapping populations. Marker clusters were identified using a minimum LOD score of 5.0 and a maximum recombination fraction (θ) of 0.35. The most likely marker order within each LG was estimated by comparing the log-likelihood of the possible orders of markers using multipoint analysis “Compare” command. The “Try” command was also used to determine the most likely placement of the unlinked markers, and subsequent orders were tested using the “Ripple” command with “Error Detection” and “Use Three Points” options enabled. The distance between neighboring markers were calculated using the multipoint analysis implemented in the “Map” command.

### Construction of Reference Consensus Genetic Map

A reference consensus genetic map was constructed using the markers mapped in ten RILs and one BC mapping populations. As peanut is an allotetraploid, deciphering the homologous versus homeologous relationships between LGs of the different component maps was necessary before constructing the consensus map. We first identified the sub-genome origin of each LG of the different component maps using a set of 58 single dose SSR markers (Table S6) that consistently amplified only one locus on the A or B sub-genomes. We then merged all LGs belonging to the same homology group with the software MergeMap [41]. In brief, LGs belonging to the same group of homology were first converted to direct acyclic graphs (DAG), which were then merged into a consensus graph on the basis of their shared vertices. Subsequently, efforts were made to resolve conflicts among the individual LGs by deleting a minimum set of marker occurrences. The result of the conflict-resolution step was a consensus DAG, which was then simplified and linearised to produce the consensus map. The final map was drawn with the help of Mapchart V 2.2 [42].

For efficient visualization of individual and consensus maps as well as their comparison, mapping data were put in the comparative mapping programme CMap version 1.01 http://www.gmod.org/cmap. This helped in assessing the congruency of marker positions and order by making a pairwise comparison among different genetic maps. Considering only the common loci existing among various genetic maps, highly conserved marker order was manifested. Subsequently, all the developed 11 individual genetic maps and the reference consensus map were aligned together in CMap.

### Conclusion

This article reports the first dense reference consensus map of the international groundnut community for wider applications in groundnut research. The consensus map provides the marker order for a maximum number of markers available in groundnut, which will be very helpful for aligning any new genetic map as well as anchoring genetic map to the future physical map. Furthermore, the reference consensus map now offers the possibility to select desirable set of markers with appropriate repeat motifs as well as PIC value that are uniformly distributed throughout the genome. In addition, marker segregation and mapping data as well as primer sequence information for as many as markers as possible have also been provided as supplementary tables that will be very useful for the groundnut community for future genetics research and breeding applications.

## Supporting Information

Figure S1 **LG wise segregation patterns of markers in each population.** In the scatter plot, markers from component mapping populations viz. RIL-1, RIL-2, RIL-3, RIL-4, RIL-5, RIL-6, RIL-7, RIL-7, RIL-8, RIL-9, RIL-10 and BC-1 are shown by blue hexagon, red square, pink triangle, orange circle sea green hexagon, bright square, plum triangle, blue circle, yellow hexagon, lavender square and violet triangle respectively.
(TIF)

Figure S2 **Classification of polymorphic markers into different ranges of PIC values.** This figure provides frequency distribution of mapped markers with variable range of PIC values.
(TIF)

Table S1 **Details of the component and the reference consensus genetic maps with marker distances.** This table provides the comparative details on mapped loci and their map distance in each LGs of component genetic maps (RIL-1, RIL-2, RIL-3, RIL-4, RIL-5, RIL-6, RIL-7, RIL-8, RIL-9, RIL-10 and BC-1) and the reference consensus map.
(XLS)

Table S2 **Summary of consensus map based on ten RILs and one BC mapping populations.** This table provides information about different homologous linkage groups used from the 11 component genetic maps for constructing the reference consensus genetic map.
(XLS)

Table S3 **Details of reference consensus genetic map with 897 marker loci based on 11 mapping populations.** This table provides BIN wise information for integrated markers along with repeat motifs, PIC values and primer sequence information.
(XLS)

Table S4 **Summary of comparative information between tetraploid cultivated reference map and with diploid groundnut maps.** This table provides comparative information on common markers between the tetraploid cultivated reference groundnut map with A-genome (Moretzsohn et al. 2005) and B-genome (Moretzsohn et al. 2009) diploid maps.
(XLS)

Table S5 **Genotyping scores for ten RILs and one BC mapping populations used to construct the reference consensus genetic map.** This table provides detailed genotyping data for all the 11 mapping populations used for the construction of reference genetic map.
(XLS)

Table S6 **List of the “A” and “B” genome specific markers mapped in the reference consensus genetic map.** This table provides list of SSR markers identified specific to A and B genomes of the tetraploid groundnut.
(XLS)

## References

[1] Beavis WD, Grant D (1991). A linkage map based on information from four F_2_ populations of maize (*Zea mays* L.).. Theor Appl Genet.

[2] Kianian SF, Quiros CF (1992). Generation of a *Brassica oleracea* composite RFLP map: linkage arrangements among various populations and evolutionary implications.. Theor Appl Genet.

[3] Gentzbittel L, Vear F, Zhang YX, Breville A, Nicolas P (1995). Development of a consensus linkage RFLP map of cultivated sunflower (*Helianthus annus* L.).. Theor Appl Genet.

[4] Hauge BM, Hanley SM, Cartinhour S, Cherry JM, Goodman HM (1993). An integrated genetic/RFLP map of the *Arabidopsis thaliana* genome.. Plant J.

[5] Sewell MM, Sherman BK, Neale DB (1999). A consensus map for loblolly pine (*Pinus taeda* L.). I. construction and integration of individual linkage maps from two outbreed three-generation pedigrees.. Genetics.

[6] Sharopova N, McMullen MD, Schultz L, Schroeder S, Sanchez-Villeda H (2002). Development and mapping of SSR markers for maize.. Plant Mol Biol.

[7] Falque M, Décousset L, Dervins D, Jacob AM, Joets J (2005). Linkage mapping of 1454 maize candidate gene loci.. Genetics.

[8] Somers DJ, Isaac P, Edwards K (2004). A high-density microsatellite consensus map for bread wheat (*Triticum aestivum* L.).. Theor Appl Genet.

[9] Varshney RK, Marcel TC, Ramsay L, Russell J, Röder MS (2007). A high density barley microsatellite consensus map with 775 SSR loci.. Theor Appl Genet.

[10] Marcel TC, Varshney RK, Barbieri M, Jafary H, de Kock MJ (2007). A high-density consensus map of barley to compare the distribution of QTLs for partial resistance to *Puccinia hordei* and of defence gene homologues.. Theor Appl Genet.

[11] Song QJ, Marek LF, Shoemaker RC, Lark KG, Concibido VC (2004). A new integrated genetic linkage map of the soybean.. Theor Appl Genet.

[12] Choi IY, Hyten DL, Matukumalli LK, Song Q, Chaky JM (2007). A soybean transcript map: gene distribution, haplotype and single- nucleotide polymorphism analysis.. Genetics.

[13] Bohra A, Saxena RK, Gnanesh BN, Saxena K, Byregowda M (2012). An intra-specific consensus genetic map of pigeonpea [*Cajanus cajan* (L.) Millspaugh] derived from six mapping populations.. Theor Appl Genet.

[14] Halward T, Stalker HT, Kochert G (1993). Development of an RFLP Linkage Map in Diploid Peanut Species.. Theor Appl Genet.

[15] Moretzsohn MC, Leoi L, Proite K, Guimaráes PM, Leal-Bertioli SCM (2005). A microsatellite-based, gene-rich linkage map for the AA genome of *Arachis* (*Fabaceae*).. Theor Appl Genet.

[16] Leal-Bertioli SC, José AC, Alves-Freitas DM, Moretzsohn MC, Guimarães PM (2009). Identification of candidate genome regions controlling disease resistance in *Arachis.*. BMC Plant Biol.

[17] Gobbi A, Teixeira C, Moretzsohn M, Guimarâes P, Leal-Bertioli S (2006). Development of a linkage map to species of B genome related to the peanut (*Arachis hypogaea* – AABB).. *In: Plant and Animal Genomes (PAG) XIV Conference*, San Diego, CA, USA. p 679..

[18] Moretzsohn MC, Barbosa AVG, Alves-Freitas DMT, Teixeira C, Leal-Bertioli SCM (2009). A linkage map for the B-genome of *Arachis* (Fabaceae) and its synteny to the A-genome.. BMC Plant Biol.

[19] Varshney RK, Bertioli DJ, Moretzsohn MC, Vadez V, Krishnamurthy L (2009). The first SSR- based genetic linkage map for cultivated groundnut (*Arachis hypogaea* L.).. Theor Appl Genet.

[20] Khedikar YP, Gowda MVC, Sarvamangala C, Patgar KV, Upadhyaya HD (2010). A QTL study on late leaf spot and rust revealed one major QTL for molecular breeding for rust resistance in groundnut (*Arachis hypogaea* L.).. Theor Appl Genet.

[21] Ravi K, Vadez V, Isobe S, Mir RR, Guo Y (2011). Identification of several small main-effect QTLs and a large number of epistatic QTLs for drought tolerance related traits in groundnut (*Arachis hypogaea* L.).. Theor Appl Genet.

[22] Sarvamangala C, Gowda MVC, Varshney RK (2011). Identification of quantitative trait loci for protein content, oil content and oil quality for groundnut (*Arachis hypogaea* L.).. Field Crops Res.

[23] Foncéka D, Hodo-Abalo T, Rivallan R, Faye I, Ndoye Sall M (2009). Genetic mapping of wild introgressions into cultivated peanut: a way toward enlarging the genetic basis of a recent allotetraploid.. BMC Plant Biol.

[24] Hong Y, Chen X, Liang X, Liu H, Zhou G (2010). A SSR-based composite genetic linkage map for the cultivated peanut (*Arachis hypogaea* L.) genome.. BMC Plant Biol.

[25] Gautami B, Pandey MK, Vadez V, Nigam SN, Ratnakumar P (2012). Quantitative trait locus analysis and construction of consensus genetic map for drought tolerance traits based on three recombinant inbred line populations in cultivated groundnut (*Arachis hypogaea* L.).. Mol Breed DOI 10.1007/s11032–011–9660–0.

[26] Sujay V, Gowda MVC, Pandey MK, Bhat RS, Khedikar YP (2012). Quantitative trait locus analysis and construction of consensus genetic map for foliar disease resistance based on two recombinant inbred line populations in cultivated groundnut (*Arachis hypogaea* L.).. Mol Breed DOI 10.1007/s11032–011–9661-z.

[27] Qin H, Feng S, Chen C, Guo Y, Knapp S (2012). An integrated genetic linkage map of cultivated peanut (*Arachis hypogaea* L.) constructed from two RIL populations.. Theor Appl Genet.

[28] Pandey MK, Gautami B, Jayakumar T, Sriswathi M, Upadhyaya HD (2012). Highly informative genic and genomic SSR markers to facilitate molecular breeding in cultivated groundnut (*Arachis hypogaea* L.).. Plant Breed.

[29] Lander ES, Green P, Abrahamson J, Barlow A, Daly MJ (1987). MAPMAKER: an interactive computer package for constructing primary genetic linkage maps of experimental and natural populations.. Genomics.

[30] Chu Ye, Holbrook CC, Ozias-Akins P (2009). Two alleles of *ahFAD2B* control the high oleic acid trait in cultivated peanut.. Crop Sci.

[31] Langridge P, Karakousis A, Collins N, Kretschmer J, Manning S (1995). A consensus linkage map of barley.. Mol Breed.

[32] Mace ES, Rami JF, Bouchet S, Klein PE, Klein RR (2009). A consensus genetic map of sorghum that integrates multiple component maps and high-throughput Diversity Array Technology (DArT) markers.. BMC Plant Biol.

[33] Hyten DL, Choi IY, Song Q, Specht JE, Carter TE (2010). A high density integrated genetic linkage map of soybean and the development of a 1536 universal soy linkage panel for quantitative trait locus mapping.. Crop Sci.

[34] Pandey MK, Monyo E, Ozias-Akins P, Liang X, Guimaraes P (2012). Advances in *Arachis* genomics for peanut improvement.. Biotech Adv.

[35] Feltus FA, Hart GE, Schertz KF, Casa AM, Kresovich S (2006). Alignment of genetic maps and QTLs between inter- and intraspecific sorghum populations.. Theor Appl Genet.

[36] Gustafson JP, Ma XF, Korzun V, Snape JW (2009). A consensus map of rye integrating mapping data from five mapping populations.. Theor Appl Genet.

[37] Fávero AP, Simpson CE, Valls JF, Vello NA (2006). Study of the evolution of cultivated peanut through crossability studies among *Ara chis ipaensis*, A. *duranensis*, and *A. hypogaea*.. Crop Sci.

[38] Foncéka D, Hodo-Abalo T, Rivallan R, Vignes H, Faye I (2012). Fostered and left behind alleles in peanut: interspecific QTL mapping reveals footprints of domestication and useful natural variation for breeding.. BMC Plant Biol.

[39] Stam P (1993). Construction of integrated genetic linkage maps by means of a new computer package JoinMap.. Plant J.

[40] Kosambi DD (1994). The estimation of map distance from recombination values.. An Eugen.

[41] Wu Y, Close TJ, Lonardi S (2008). On the accurate construction of consensus genetic maps.. In: Proceedings of LSS Computational Systems. *Bioinformatics Conference*, 26–29 August 2008, Stanford Edited by: Peter Markstein p285–296..

[42] Voorrips RE: Mapchart (2002). software for the graphical presentation of linkage maps and QTLs.. J Hered.

