# Supplementary figures and images for: An International Reference Consensus Genetic Map with 897 Marker Loci Based on 11 Mapping Populations for Tetraploid Groundnut (Arachis hypogaea L.)
Source: PLoS One. 2012 Jul 18;7(7):e41213. doi: 10.1371/journal.pone.0041213 (PMC3399818; doi:10.1371/journal.pone.0041213)

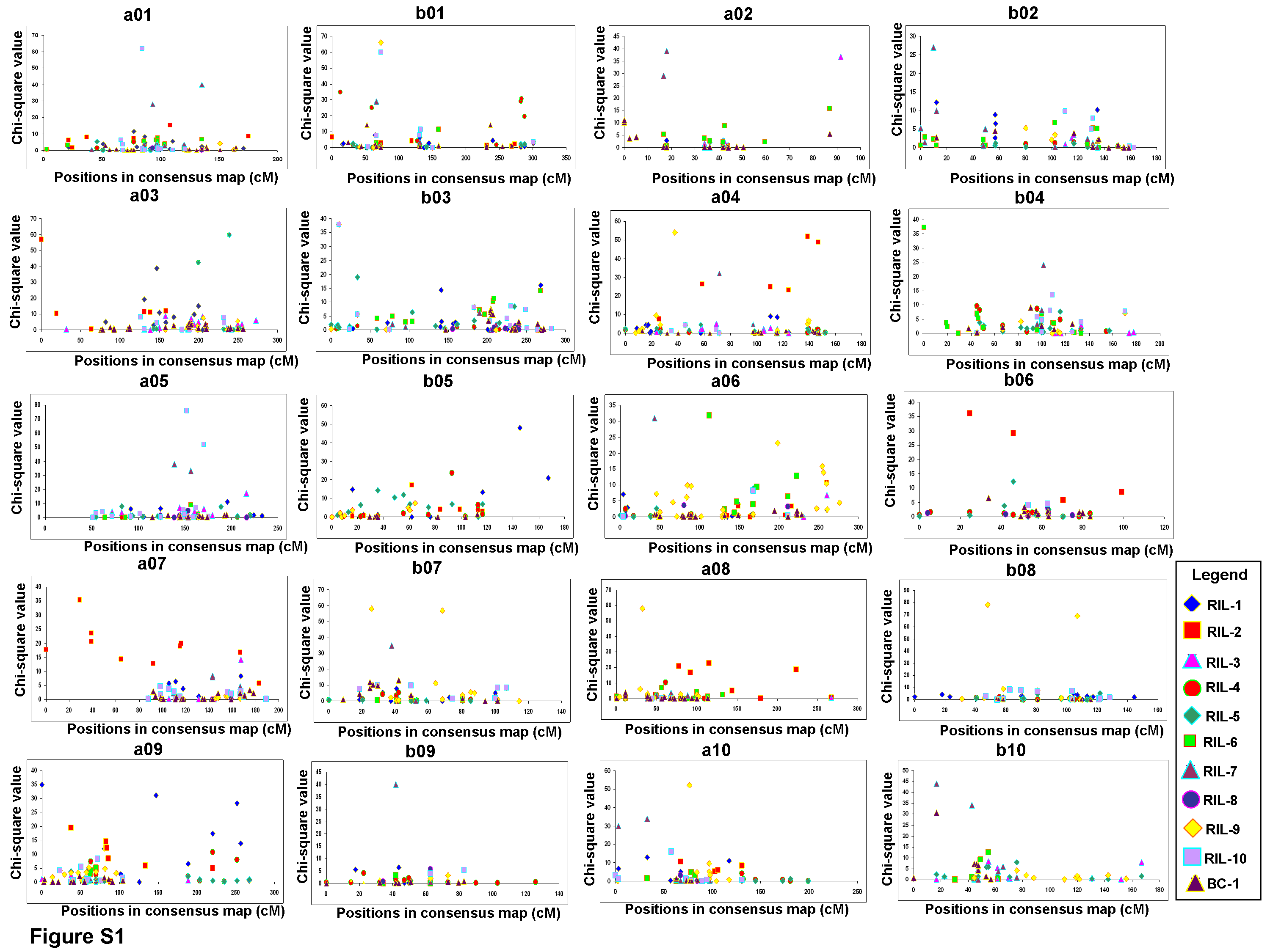

Supplement: Figure S1 — LG wise segregation patterns of markers in each population. In the scatter plot, markers from component mapping populations viz. RIL-1, RIL-2, RIL-3, RIL-4, RIL-5, RIL-6, RIL-7, RIL-7, RIL-8, RIL-9, RIL-10 and BC-1 are shown by blue hexagon, red square, pink triangle, orange circle sea green hexagon, bright square, plum triangle, blue circle, yellow hexagon, lavender square and violet triangle respectively. (TIF) [file pone.0041213.s001.tif]

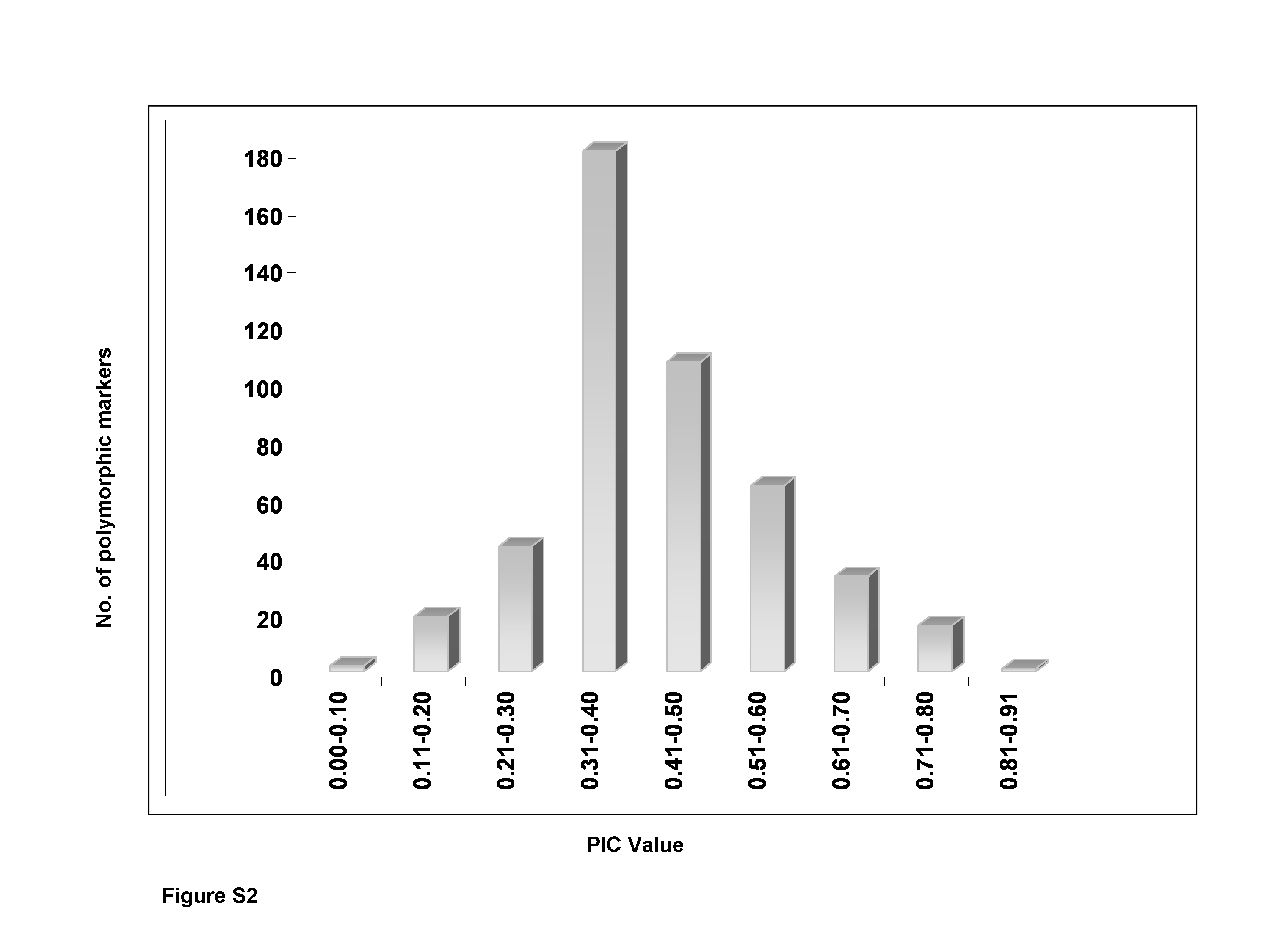

Supplement: Figure S2 — Classification of polymorphic markers into different ranges of PIC values. This figure provides frequency distribution of mapped markers with variable range of PIC values. (TIF) [file pone.0041213.s002.tif]
